# A wet-filtration-zipping approach for fabricating highly electroconductive and auxetic graphene/carbon nanotube hybrid buckypaper

**DOI:** 10.1038/s41598-018-30009-4

**Published:** 2018-08-15

**Authors:** Shashikant P. Patole, Muhamad F. Arif, Rahmat A. Susantyoko, Saif Almheiri, S. Kumar

**Affiliations:** 1 Department of Mechanical and Materials Engineering, Khalifa University of Science and Technology, Masdar Institute, Masdar City, P.O. Box 54224, Abu Dhabi, UAE; 2 Department of Physics, Khalifa University of Science and Technology, P.O. Box 127788, Abu Dhabi, UAE

## Abstract

A combination of carbon nanotubes (CNT) and graphene in the form of macroscopic hybrid buckypaper (HBP), exhibits a unique set of properties that can be exploited for many emerging applications. Here, we present a simple, inexpensive and scalable approach for the synthesis of highly conductive auxetic graphene/CNT HBP via wet-filtration-zipping and demonstrate the electrical, electrochemical and mechanical performance (tensile, mode I and mode III fracture) of synthesized HBP. An overall increase in electrical conductivity of 247% is observed for HBP (50 wt.% graphene and 50 wt.% CNT) as compared to BP (100 wt.% CNT) due to effective electronic percolation through the graphene and CNT. As a negative electrode for lithium-ion batteries, HBP shows 50% higher gravimetric specific capacity and 89% lower charge transfer resistance relative to BP. The graphene content in the HBP influences the mechanical performance providing an auxetic structure to HBP with large negative Poisson’s ratio. The facile green-chemistry approach reported here can be readily applied to any other 1D and 2D materials and solves key challenges associated with existing buckypaper manufacturing methods. The potential of the synthesis method to integrate with current cellulose paper manufacturing technology and its scalability demonstrate the novelty of the work for industrial scale production.

## Introduction

Carbon nanotubes (CNT) and graphene consist of *sp*^2^ bonded carbon atoms arranged in a honeycomb lattice structure and possess exceptionally high mechanical properties, and electrical and thermal conductivities^1^. The origin of exceptionally high Young’s modulus, 1.2 TPa, electrical conductivity, 10^6^ S/m, and thermal conductivity, 3000 W/mK, are due to the bond strength of *sp*^2^ hybridized carbon, out of plane π-orbitals with unique band structure, and phonon transport, respectively^2–6^. They are highly sought for advanced energy, structural and electronic applications^7–9^. Due to their microscopic nature, they are difficult to handle and therefore need a macroscopic assembly for real world applications. Carbon nanotube papers (commonly known as buckypaper, BP)^10–37^, graphene papers, graphene oxide (GO) papers, reduced graphene oxide (rGO) papers, GO/rGO-CNT hybrid BP (HBP), and graphene-CNT HBP are some of the macroscopic assemblies which represent the outstanding nanoscale material properties in macroscale form, leading to the creation of strong, foldable, auxetic and highly conductive lightweight materials. These assemblies, in particular, HBP combines 1 dimensional (1-D) properties of CNT and 2 dimensional (2-D) properties of graphene in a single 3-dimensional (3-D) macroscopic structure. Moreover, these assemblies are important in various applications, such as in water and air purification^38,39^, energy storage^16,40^, sensors^41,42^, aviation^43,44^, medical devices^45,46^, composites^47,48^ etc. Another interesting aspect of BP/HBP is that the realignment of entangled nanotubes during stretching gives a large negative Poisson’s ratio, and such materials are usually termed auxetic materials. Auxetic materials have various applications, such as press-fit fasteners, curved sandwich panels, flexible impact buffers, soundproof materials, and so on^10,12,13^. Recent progress in multi-walled CNT (MWCNT) and graphene production provides these materials at scalable quantity^49,50^. Concomitantly, the manufacturing of BP/HBP also needs a low-cost, simple, scalable procedure to convert the CNT and graphene powder into BP/HBP.

The HBP production is new and mostly utilizes the existing BP production methods. Four major BP production methods have hitherto been reported in the literature. In the 1^st^ method, BP is directly produced during CNT growth by chemical vapor deposition - also known as CVD method^21^ or post-CNT synthesis using dry techniques of shear pressing, alcohol drenching and pressing, domino pushing and CNT drawing. A disadvantage of this technique is that the resulting BP is typically not thick. Moreover, the production process is not roll-to-roll. In the 2^nd^ method, BP is produced by powder compaction and frit-compression. However, the powder compaction requires a mold, thus the created BP may not be flexible nor foldable. Despite the thicker BP compared to 1^st^ method, frit-compression technique requires a relatively high cost membrane which hinders scalability. The 3^rd^ method involves techniques such as drop casting^28,30^, rod coating^27^, air spraying, and tape-casting^15,26^. Yun *et al*. described the fabrication of BP using tape-casting on a mold followed by vacuum oven heating^15^. The need of mold as well as vacuum heating process may hamper this method’s scalability. Moreover, it is problematic to detach the CNT layer/film from the supporting substrate to get freestanding BP due to low surface energy of CNT. Recently, to overcome this problem, Susantyoko *et al*. developed a surface-engineered tape cast method to manufacture flexible, freestanding, and foldable BP on a roll-to-roll system without the usage of mold^16^. However, a special need for surface-engineered conveyor belt limits its use in the laboratories. The 4^th^ method is the membrane filtration^14–38^, which can be effortlessly installed in laboratories. Despite the common usage of this method, challenges of the membrane-filtration method include membranes with a relatively high-cost; requirement of hazardous chemicals; requirement of vacuum; requirement of high pressure; long filtration time due to the high fluid flow resistance (particularly for thick BP); relatively small BP diameter (typically diameter ≤ 9 cm); and relative non-uniform BP thickness. Because membrane filtration has a low-throughput, it may not be a potential candidate for mass production of BP. We solved these challenges using environment-friendly water based wet-filtration-zipping. In this method water with the surfactant is used to disperse the hydrophobic MWCNT and graphene to obtain their well dispersed solution. We use inexpensive cellulose filter papers with relatively large pore size (11 µm) and thickness (180 µm) to obtain MWCNT and graphene sediment on it by gravity. Further, MWCNT-graphene sediment was zipped due to elasto-capillary densification and thus a freestanding BP/HBP^51–54^ is formed. Our process has the potential to integrate with the current industrial paper manufacturing technology. Other advantages are scalability, uniform thickness (at micron level), and potential of the synthesis route to accommodate any other 1D and 2D materials (e.g. water soluble 1D and 2D materials).

Another aim of this work is to study the electrical, electrochemical and mechanical performance of BP/HBP. We have demonstrated potential application of HBP as an anode for lithium-ion batteries. The anode is one of the most important part of lithium-ion batteries since the anode characteristics influence their electrochemical performance. Incumbent anode utilizes graphitic carbon because of its advantageous features, such as the excellent electrical conductivity, beneficial hierarchical structure for the intercalation of lithium ion, low-cost and abundant availability^55^. However, there are few limitations of graphitic anode such as low specific capacity and mediocre rate capability, warranting further research to advance the performance of carbon-based anode materials. CNT and graphene are promising candidates for large capacity lithium-ion batteries due to their excellent conductivity as well as stability. In addition to the high specific surface area, the two sides of graphene sheets, and internal as well as external walls of CNT can be fully utilized to store lithium ions to form LiC_3_, thereby raising anode theoretical specific capacity (744 mA h g^−1^) to more than twofold of the graphite (has a lower surface area and forms LiC_6_ during lithiation) specific capacity (372 mA h g^−1^). Consequently, the right selection of material and appropriate architectural design modification are crucial for excellent battery performance. It is expected that the combination of CNT and graphene in HBP will improve the anode performance. Therefore, the anodic performance of binder free HBP is evaluated using cyclic voltammetry, electrochemical impedance spectroscopy and galvanostatic cycling measurements with lithium metal as the counter electrode.

As such, without any chemical bonding and binder, these free-standing self-assemblies are held together due to entanglement and weak van der Waals interaction. It is important to understand the tensile and fracture behavior of such assemblies. Therefore we also focused on evaluating their tensile properties and fracture resistance in mode I and mode III, and identified the corresponding failure mechanisms.

## Results and Discussion

**Figure 1:**
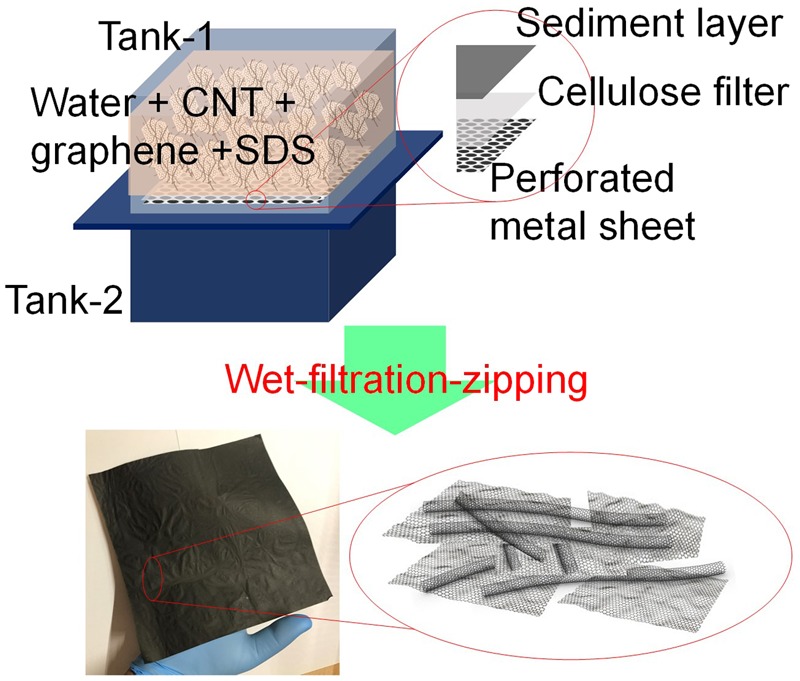
Schematic showing the preparation of hybrid buckypaper (HBP) by the wet-filtration-zipping method. A well dispersed-MWCNT and graphene crystals (GC) in water is filtrated to obtain the HBP.

MWCNT and graphene crystals (GC) produced at industrial scale are used for the fabrication of buckypaper (BP) and hybrid buckypaper (HBP)^49,50^. MWCNT are synthesized by a continuous atmospheric CVD system with a glass fiber fabric substrate^49^. The diameter of MWCNT is within 10–30 nm range with 3–10 walls (see Supporting Information Figure S1). It is observed that in most of the MWCNT the inner walls are intact, but the outer walls are attached to the other MWCNT making it an outstanding candidate for the buckypaper where the covalently bonded and entangled network of MWCNT is highly desirable for the electronic percolation and improved mechanical performance. GC are produced by the intercalation expansion-exfoliation process, which gives unique wrinkled and crumpled morphology to the graphene flakes. The lateral spread of the graphene flakes in GC is in the range of 10–300 µm^50^. Overall the samples contain less than 10 layers of graphene. An aberration-corrected HR-TEM shows hexagonal honeycomb lattice with clearly distinct carbon atoms of graphene (Supporting Information Figure S1). It should be noted that GC are highly crystalline in nature due to its unique production method which does not allow oxidation of graphitic lattice. The high crystallinity is also revealed via Raman (negligible D band indicating defect free graphene lattice) and thermogravimetric analyses (TGA) (85% mass remained at 1000 °C) (Supporting Information Figure S2). Therefore GC are also distinct from the rGO/GO where a lot of missing carbon atoms and the presence of oxygen deteriorate its crystallinity and conductivity. It is expected that the combination of 1D 110–160 µm MWCNT and highly-crystalline 2D GC will enhance the conductivity of combined assembly. In order to form an assembly of MWCNT and GC, they were first dispersed in water with the help of surfactant and tip-sonication. A well-dispersed solution is then poured into the metal tank 1 of the indigenously fabricated wet-filtration assembly as shown in Fig. 1. The details of the dispersion and wet-filtration assembly are provided in the Methods Section. In short, the wet-filtration assembly consists of two metallic tanks separated by a perforated metal sheet with a filter paper on it. The wet filtration procedure allows separating the sediment of MWCNT and graphene on the top of filter paper. As the pore size of filter paper is 11 µm, it does not allow MWCNT and GC to pass through it. After draining the water from the tank 1 by gravity, the perforated sheet with filter paper and sediments were separated from the filtration assembly and dried overnight in a heating oven at 90 °C at atmospheric pressure. After drying, a free-standing BP/HBP is easily peeled off from the filter paper. A drastic reduction in the thickness of the sediment layer was observed after drying. During the drying process, the removal of water molecules from the gaps of MWCNT/GC networks forced the adjacent MWCNT/GC to come close enough to form a dense assembly. The elasto-capillary effect due to the excessive surface tension is mainly responsible for such zipping. As a result, few millimeter thick sediment layer is zipped into the few micrometer thick paper. The overall assembly consists of densely packed and entangled GC embedded MWCNT network with a density of 1 g/cc (graphite bulk density is 2.26 g/cc). In the previous studies, a similar analogy is used to form CNT wafers and CNT micropillars^53,54^. Hayamizu *et al*. used isopropyl alcohol to wet the CNT forest and then dried it to obtain the densely packed CNT wafers^53^. De Volder *et al*. used acetone to densify the CNT micropillars^54^. The evaporation of organic solvent forced the adjacent CNT to come closer and solidify. Here, we do not use any organic solvent, rather used only water and observed the same zipping effect. The surface tension of water (72.8 mN/m) is much higher than that of the ethanol (23 mN/m) and acetone (25.2 mN/m) and therefore higher densification is expected in the case of water. It is expected that the weak van der Waals forces, entangled network of MWCNT and covalent bonding in MWCNT shared walls are responsible for holding the whole BP/HBP assembly. Moreover, MWCNT and GC are not functionalized and therefore other chemical bonding such as hydrogen and hydroxyl is absent. The lateral dimensions of BP/HBP depend on the size of the metal tank whereas the thickness of BP/HBP depends on the amount of MWCNT and GC thus allowing scalability over lateral size and thickness (Supporting Information Figure S3). Moreover, the wet-filtration-zipping method can be integrated into the continuous paper manufacturing process (Supporting Information Figure S4). In a typical synthesis, 1 l water with 1 g of MWCNT/GC produces 80 µm thick and 18 cm × 18 cm BP/HBP (Fig. 1). Compression of BP/HBP results in reduction in thickness (10–70%) resulting in increased density (Supporting Information Figure S5). Both BP and HBP are lightweight and flexible. The appearance of BP is completely black whereas the HBP appears grey and sparkling due to the presence of GC in it (Supporting Information Figure S6).

**Figure 2:**
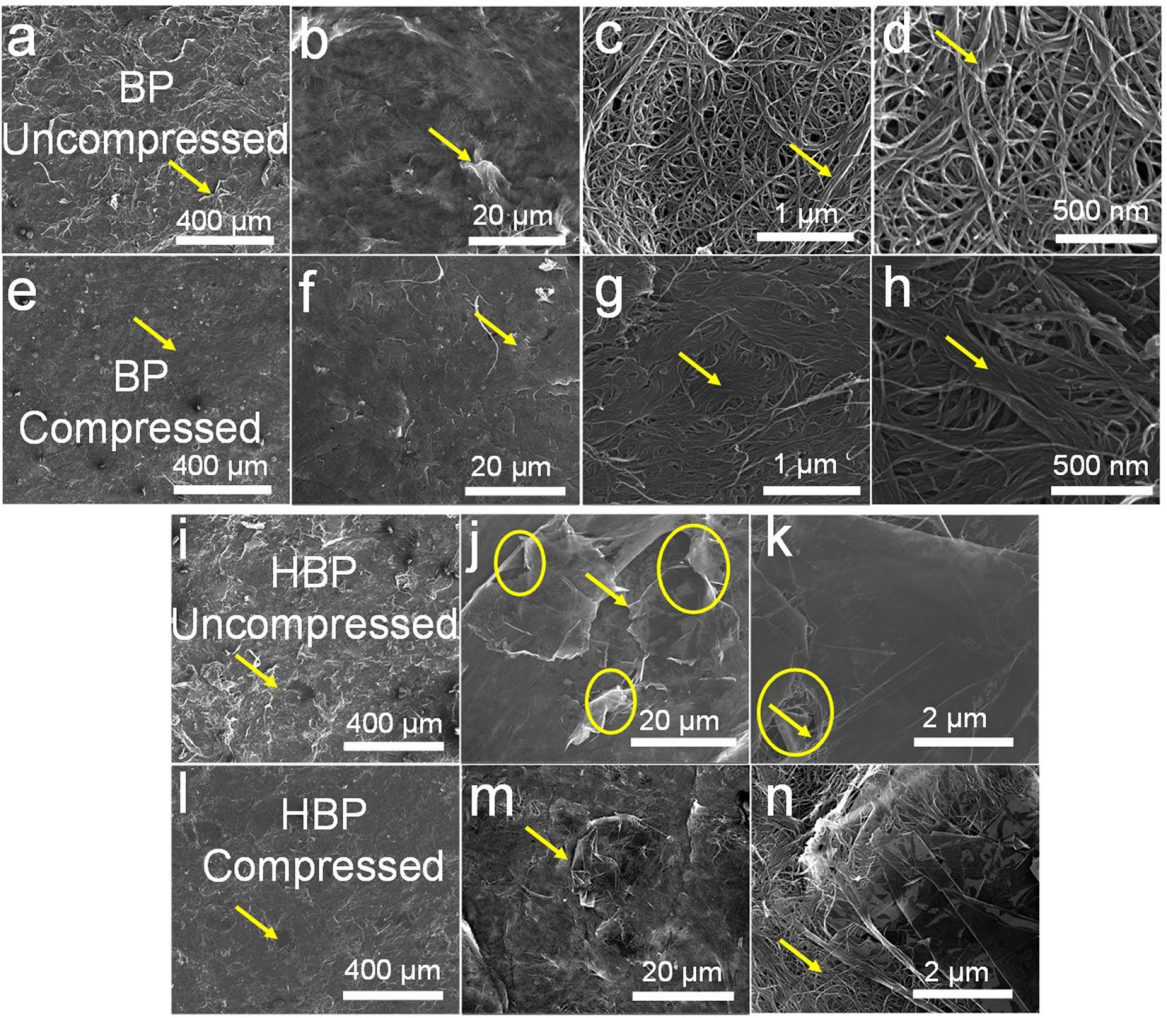
SEM images showing surface morphology of (**a**–**d**) as prepared (uncompressed) BP; (**e**–**h**) compressed BP; (**i**–**k**) uncompressed HBP (GC = 50 wt.%); and (**l**–**n**) compressed HBP. A 25 MPa pressure was applied to compress the samples. The arrows in figures (**a**–**h**) indicate the bundled regions undergoing flattening after the compression, the arrows in figures (**i**–**j**) indicate the distinguishable GC flakes in the HBP, the arrows in Figures (**k** and **n**) indicate the flattening of MWCNT networks attached to the GC flakes. The circles in the Figures (**j** and **k**) indicate the voids in in the HBP due to crumpled GC.

Surface morphology of as-prepared (uncompressed) and compressed BP observed in SEM is shown in Fig. 2. The MWCNT bundles (marked by the arrows in Fig. 2a,b) give wavy hump-like features to the BP surface. It should be noted that in the as-received MWCNT samples, MWCNT are entangled and are densely packed within the individual cakes. After several hours of ultra-sonication, it was observed that most of the MWCNT share their outer walls with other MWCNT making it difficult to isolate a single MWCNT (Supporting Information Figure S1). In this scenario, it is expected that such MWCNT bundles in the filtrate sediment contribute to the wavy hump-like features. At higher magnification (Fig. 2c,d), individual MWCNT porous network with porosity10–100 nm is observed. Apart from the thin bundles (marked by the arrow), MWCNT are well entangled giving an interconnected network of MWCNT. A noticeable difference in the surface morphology is observed for the compressed BP samples (Fig. 2e–h). After compression, the humps of MWCNT bundles are flattened as marked by the arrows in Fig. 2e and f. It is observed that the BP become glossy after compression due to the higher reflection of light from the compressed flattened surface. At higher magnification (Fig. 2g and h), a flattened MWCNT network and bundles are observed. The overall porosity of 10–100 nm before compression is reduced to 1–10 nm after compression. Thus the compression has reduced the inter-tube voids making it a denser structure than before. The observed reduction in the thickness and increase in the density are 25% and 33% respectively.

As prepared (uncompressed) and compressed HBP show a distinct surface morphology as compared to BP (Fig. 2i–n). Apart from MWCNT bundles, GC flakes are clearly observed contributing to the overall wavy and bumpy surface of HBP (marked by arrows in Fig. 2i and j). Individual GC are spread laterally rather than vertically into the HBP due to gravity. Moreover, the spread GC sheets are covered by the MWCNT network making it a true 3D hybrid assembly of 1D MWCNT and 2D graphene. The as-received GC are crumpled in nature with the wrinkled surface which adds extra voids into the HBP marked by circles in Fig. 2j and k. After compression, the humps are flattened as shown in Fig. 2l. The individual crumpled GC flatten in such a way that the voids are minimized (Fig. 2m). The MWCNT networks also undergo densification similar to BP (as marked by an arrow in Fig. 2n). The observed overall reduction in the thickness and increase in the density are 23% and 29% respectively. Compression of HBP offers more flattened, denser and more interconnected network of MWCNT and GC compared to the uncompressed samples.

**Figure 3:**
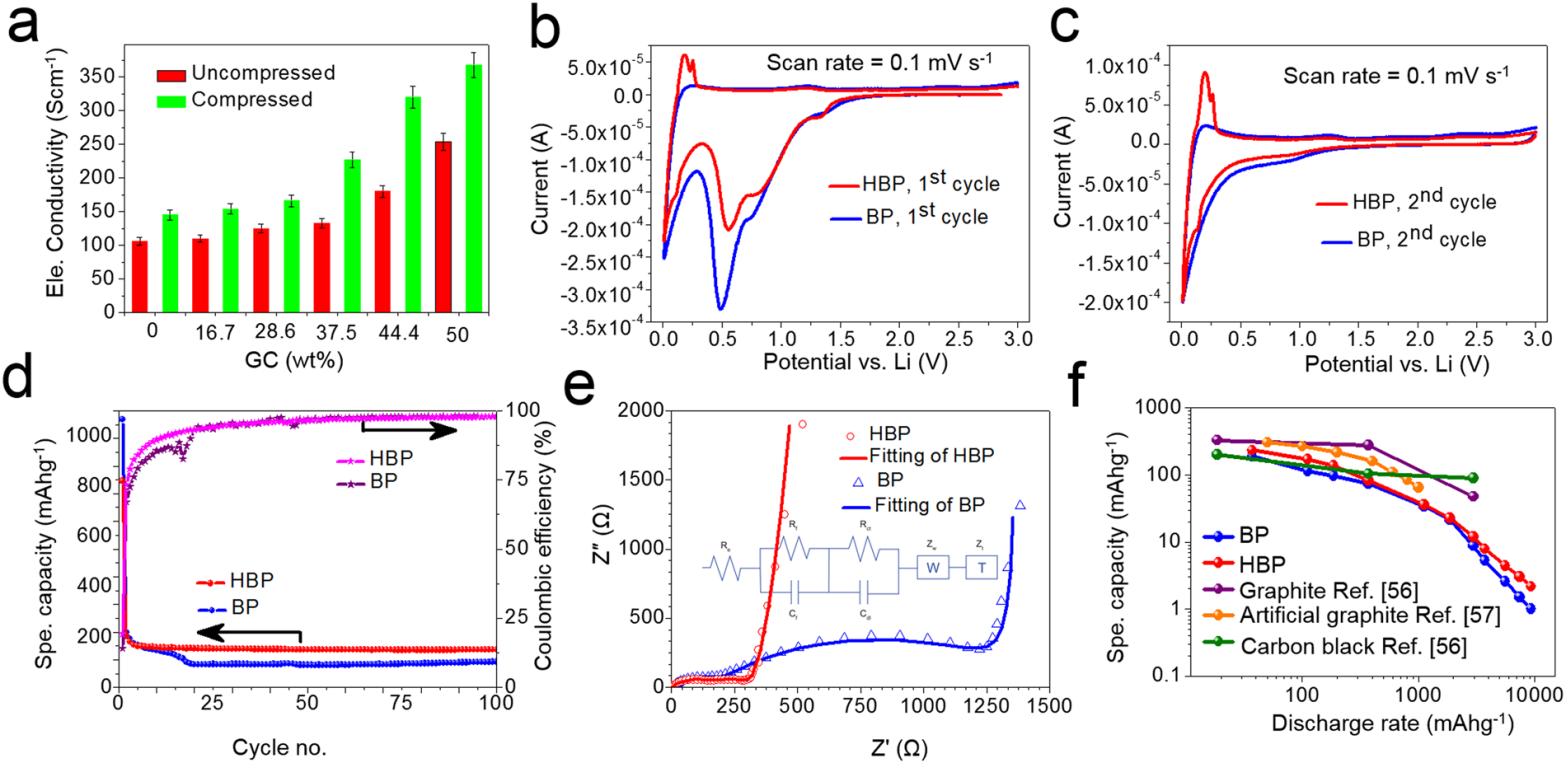
Electrical and electrochemical performance: (**a**) Effect of compression and graphene content in HBP on the electrical conductivity. (**b**) 1^st^ cycle and (**c**) 2^nd^ cycle cyclic voltammetry of HBP (50 wt.% GC) and BP sheet at a scan rate of 0.1 mV s^−1^. (**d**) Cycle test of HBP and BP for 100 cycles at 186 mA g^−1^ rate. (**e**) Impedance spectroscopy analysis: the data, fitting and equivalent circuit of HBP and BP. (**f**) The rate capability of HBP and BP at various discharge rates.

The GC content and compressed HBP show an ameliorating effect on the electrical conductivity (Fig. 3a). The electrical conductivity of BP is 106 S/cm while the electrical conductivity of the compressed BP is 145 S/cm. A 50 wt.% GC content HBP shows the electrical conductivity of 254 S/cm which increases up to 368 S/cm after compression. An average of 35% improvement in the electrical conductivity of BP after compression is observed whereas 247% improvement in the electrical conductivity is observed for HBP with 50 wt.% GC. The individual MWCNT and graphene have very high electrical conductivity ~10^2^–10^6^ S/cm. The difference in the electrical properties of our HBP and the MWCNT/graphene is due to the discontinuity between the individual MWCNT/GC and the weak electrical conductivity between the contacts of MWCNT–MWCNT, MWCNT–GC, GC-GC. In comparison with the electrical properties of published work of BP/HBP, our BP exhibits properties similar to BP with randomly oriented CNT, and our HBP with HBP comprising self-assembly of CNT-graphene. However, our BP/HBP exhibits marginal properties compared to the BP with oriented CNT and HBP with chemically bonded CNT-graphene. The difference in these properties comes from various parameters such as graphene type (GO, rGO), CNT diameter and length, alignment, density, graphene flake size, chemical treatment, processing condition, fabrication method, nature of bonding between CNT and graphene, etc.

Figure 3b,c show the cyclic voltammetry of HBP (50 wt.% GC + 50 wt.% MWCNT) and BP (100 wt.% MWCNT). During the first cathodic scan, both HBP and BP had peaks in the potential range between 0.25 V and 1.5 V, which are attributed to the electrolyte decomposition and formation of solid electrolyte interphase (SEI). These cathodic peaks disappeared in the subsequent cycle, see Fig. 3b. Figure 3c shows HBP has less irreversible capacity due to electrolyte decomposition compared to BP sheet. The cathodic peaks between 0.01 V and 0.25 V were attributed to the insertion of lithium-ion. During the first anodic scan, HBP had sharp peaks of 0.2 V and 0.26 V, attributed to lithium-ion deintercalation from graphitic structure. Figure 3d shows the cycle test of HBP and BP samples. HBP at 186 mAg^−1^ discharge rate had 1^st^, 2^nd^, 50^th^ and 100^th^ cycle specific capacity of 821.4, 204.8, 149.6 and 149.7 mAhg^−1^, respectively. BP has specific capacity of 1065.5, 218.8, 88.8 and 101.9 mAhg^−1^ at 1^st^, 2^nd^, 50^th^ and 100^th^ cycle, respectively. The superior specific capacity of HPB after cycles can be attributed to secondary lithium-ion storage mechanism in the disordered stacks of graphene sheets. The specific capacity drop from 1^st^ cycle to 2^nd^ cycle of 616.5 mAhg^−1^ of HBP is lower than that of BP of 846.653 mAhg^−1^. Electrochemical impedance spectroscopy (EIS) was performed and the data was fitted using equivalent circuit in Fig. 3e. The results showed that charge transfer resistance of HBP of 127 Ω is smaller than that of BP of 1204 Ω, which indicated fast reaction kinetics of HPB consistent with the superiority of HBP compared to BP in terms of rate capability (Fig. 3f). HBP has a larger specific capacity in all cases when tested at various discharge rates compared to pristine BP. Moreover, the specific capacity of HPB is comparable to that of graphite and carbon black anodes^56,57^. However, being a freestanding structure, the HPB does not have dead weights from metal current collectors, which set it apart from references^56,57^. In short, the right selection of material, and appropriate architectural design modification are crucial for excellent performance of battery. As a result, the combination of MWCNT and graphene in HBP improves the anode performance.

**Figure 4:**
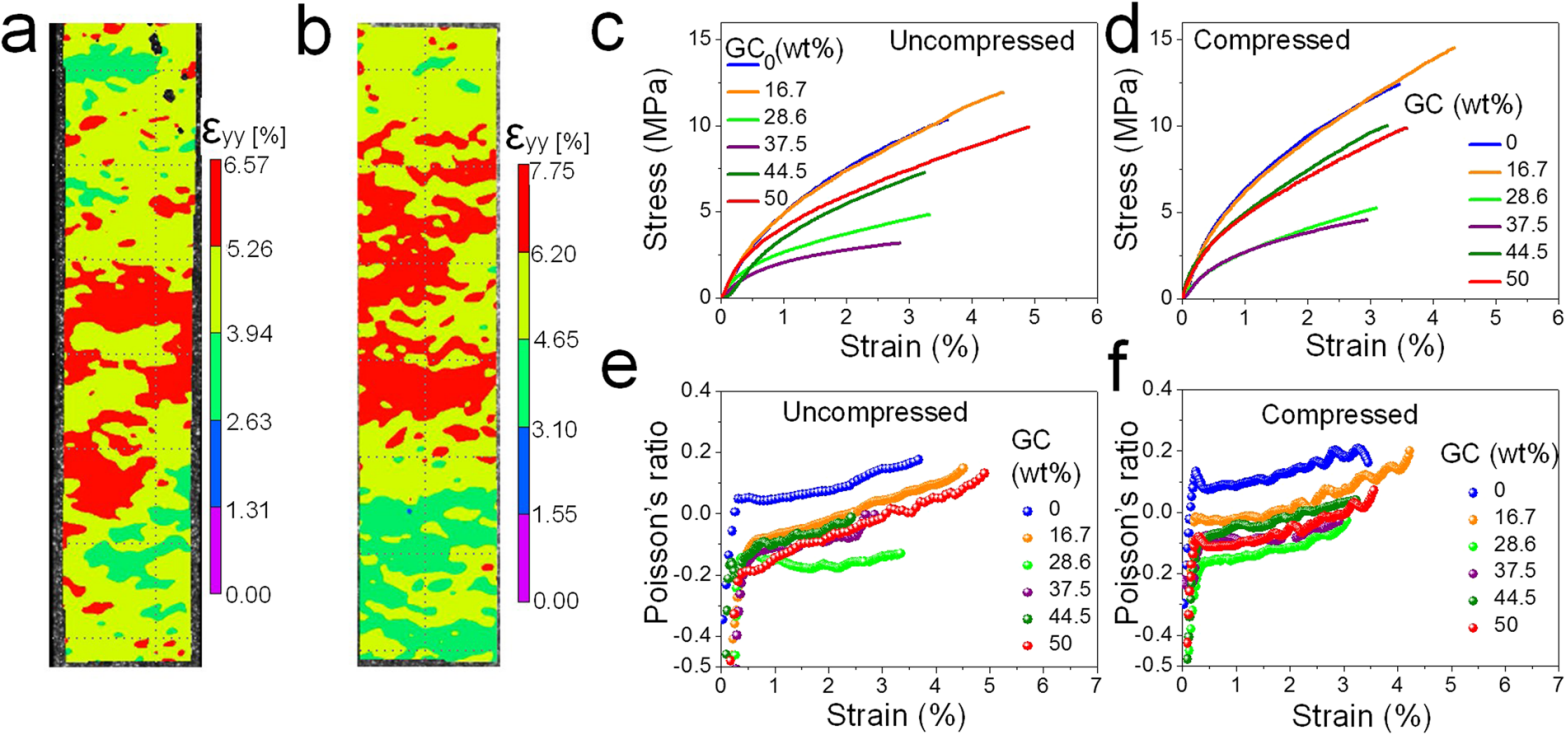
The effect of GC content in the HBP on mechanical properties: Digital image correlation (DIC) images showing strain distribution at maximum load of (**a**) BP and (**b**) HBP. The representative tensile stress-strain curve of (**c**) as prepared (uncompressed) samples, and (**d**) compressed samples. The evolution of Poisson’s ratio with strain of (**e**) uncompressed, and (**f**) compressed samples.

BP and HBP are highly desirable next generation carbon-based materials which have potential to replace the carbon fibers in multifunctional composites. It incorporates the outstanding properties of 1D CNT and 2D graphene. In this regard, mechanical properties of both BP, and HBP are important. Tensile response of BP and HBP are shown in Fig. 4. Evolution of surface strain field was measured by digital image correlation (DIC). The zone evaluated for the DIC can be seen in Fig. 4a,b. The strain reported in Fig. 4c–f is the average value of the evaluated zone. The DIC results show the strain field evolution as the load increases during testing (Supporting Information Movies S1 and S2). Non-uniform strain field generated in BP and HBP samples indicates the randomly oriented MWCNT in the BP and GC-MWCNT in the HBP. This is due to the local MWCNT/GC microstructure, such as the fiber and fiber-flake entanglement, wall sharing, flake over flake shearing, branching and crosslinking. The strain measured by DIC provides reliable results since errors associated with strain due to clamping effect can be discarded^58^.

Figure 4c,d show the stress-strain response of uncompressed and compressed HBP under tensile loading for various GC content. The corresponding mean value and standard error of Young’s modulus, maximum stress, strain at break, and toughness are provided in Table 1. It is clearly observed that the addition of GC into the MWCNT deteriorated the mechanical performance of HBP (Fig. 4c). This peculiar trend shows a decrease in the mechanical performance up to 37.5 wt.% GC content. However, further addition of GC up to 50 wt.% improves the mechanical performance. Generally, our BP and HBP have tensile performance similar to BP with randomly orientated CNT^10,38,59,60^. The compression further improves the mechanical performance of BP and HBP (Fig. 4d) as compression increases the fiber density, minimizes the voids, improves the CNT-graphene interaction. One can see that alteration in pore diameter and density of BP/HBP due to pressing force can significantly change the mechanical performance. The pressing force improves the van der Waals interaction between random MWCNT-graphene networks giving rise to improved mechanical properties. The pressure minimizes the distance between MWCNT, MWCNT-graphene, and graphene-graphene (Fig. 2). Since the MWCNT and graphene are randomly oriented, the improved van der Waals bonding occurs at the junctions between neighboring MWCNT, crossover lengths of MWCNT-graphene, and graphene-graphene overlapping areas. Nonetheless, the improvement in mechanical properties is not up to the mark of CNT and graphene due to the fact that only microscopic van der Waals forces and entanglements between CNT are contributing to the overall mechanical properties rather than atomic *sp*^2^ carbon bonding. The potential of *sp*^2^ carbon bonding is not utilized due to the lack of any covalent bonding at the crossover junctions. It is expected that the covalent bonding between CNT-CNT and CNT-graphene may utilize the true strength of CNT and graphene in HBP to yield a material with high strength and high toughness^61^. The Poisson’s ratio can be specified as $\nu =-\,\frac{d{\varepsilon }_{x}\,/\,d\sigma }{d{\varepsilon }_{y}\,/\,d\sigma }$ where ${\varepsilon }_{x}$ and ${\varepsilon }_{y}$ are the transverse and axial strains, respectively, and $\sigma$ is the axial stress. The observed Poisson’s ratio for uncompressed and compressed HBP for various GC content is shown in Fig. 4e,f, respectively. It can be seen that the Poisson’s ratio is initially negative for all samples and then stabilizes as the strain increases. The MWCNT realignment in the axial loading direction leads to a stable positive Poisson’s ratio. In the stable strain region, for uncompressed and compressed samples, the Poisson’s ratio of the BP (GC = 0 wt.%) is positive whereas for the HBP, it is negative. The presence of GC flakes in the MWCNT network aids higher transverse expansion with load and thus results in negative Poisson’s ratio.

**Table 1:** Tensile properties of BP/HBP samples. The ± sign indicates the standard error.

Amount (%)		Young’s modulus (MPa)		Maximum stress (MPa)		Strain to failure (%)		Toughness (kJ.m^−3^)	
GC	CNT	Uncompressed	Compressed	Uncompressed	Compressed	Uncompressed	Compressed	Uncompressed	Compressed
0	100	684 ± 33.0	846 ± 39.7	10.7 ± 0.61	13.5 ± 0.91	4.06 ± 0.23	3.53 ± 0.18	286 ± 27.9	387 ± 41.2
16.7	83.3	616 ± 16.7	1009 ± 49.3	11.3 ± 0.51	14.7 ± 0.52	4.67 ± 0.33	4.20 ± 0.33	316 ± 31.0	394 ± 49.0
28.6	71.4	321 ± 10.0	542 ± 16.7	4.8 ± 0.23	5.1 ± 0.35	3.26 ± 0.16	3.20 ± 0.27	106 ± 12.4	101 ± 13.3
37.5	62.5	328 ± 14.5	543 ± 21.6	2.9 ± 0.15	3.9 ± 0.21	2.90 ± 0.20	2.33 ± 0.20	57 ± 7.24	90 ± 10.1
44.5	55.5	691 ± 29.1	752 ± 29.9	6.6 ± 0.22	9.2 ± 0.38	2.41 ± 0.25	3.36 ± 0.25	105 ± 12.9	205 ± 22.2
50	50	486 ± 22.5	907 ± 44.0	9.8 ± 0.36	9.7 ± 0.51	5.01 ± 0.26	3.45 ± 0.29	347 ± 37.0	222 ± 27.1

**Figure 5:**
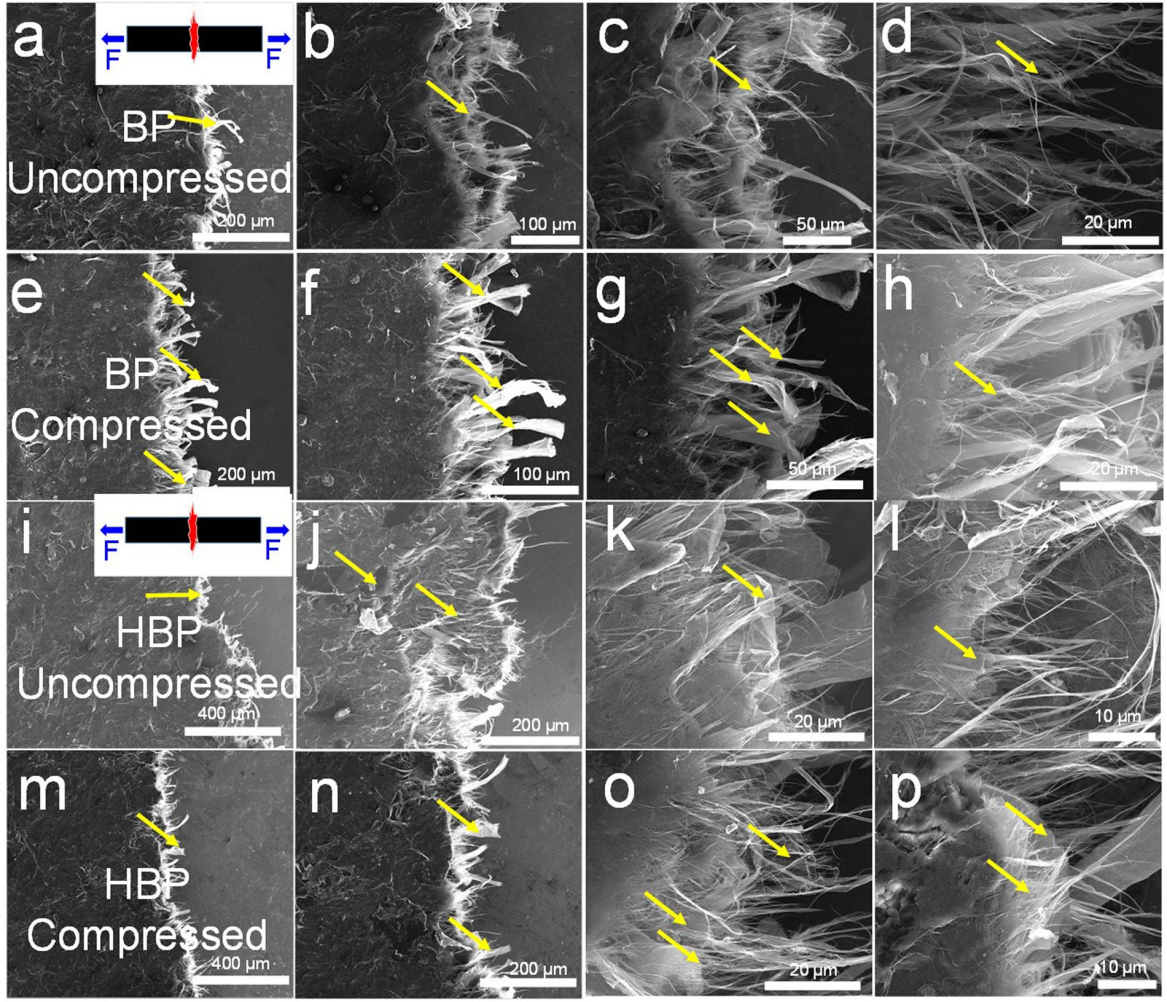
The post tensile test analysis of BP and HBP specimens: SEM images showing the fracture region of (**a**–**d**) uncompressed BP, (**e**–**h**) compressed BP, (**i**–**l**) uncompressed HBP (GC = 50 wt.%), and (**m**–**p**) compressed HBP. The pull out regions in the uncompressed and compressed BP can be distinguished by the diluted MWCNT and dense (bundled) MWCNT as marked by the arrows in the figures (**a**–**h**) and the pull out regions in the uncompressed and compressed HBP can be distinguished near the graphene flakes as marked by the arrows in the figures (**i**–**h**). Inset in a, and i shows the tensile force direction and fractured region of the specimen.

In order to understand the role of MWCNT and GC on the mechanical performance of BP and HBP, post-tensile test specimens were analyzed under the SEM. Figure 5 shows the fracture region of uncompressed (Fig. 5a–d) and compressed (Fig. 5e–h) BP samples. The direction of applied tensile force, $F$ is shown by the arrows in the inset of Fig. 5a. The pulled out MWCNT in the direction of force are clearly observed in both samples. The MWCNT density decreases in the pull out direction. At the same time, more aligned MWCNT in the pull out direction are observed. It clearly demonstrates that the entangled network of MWCNT is stretched during the tensile test. The observed stress-strain response is a result of pull out of MWCNT from the entangled network rather than breaking of individual MWCNT. This also justifies the observed lower Young’s modulus and tensile strength as compared to those of individual CNT and graphene. The individual CNT and graphene can have Young’s modulus of 1–1.2 TPa and strength of 125–150 GPa^3,9^. Relative to the uncompressed samples wherein diluted MWCNT bundles are observed in the pull out region, the compressed samples show (Fig. 5e–h) dense MWCNT bundles. Moreover, the pulled out bundles are also aligned in the direction of the tensile force. The compression improves the MWCNT density which helps to hold the MWCNT network, resulting in better mechanical performance compared to the uncompressed samples (Fig. 4c,d).

The fracture regions of HBP with 50 wt.% MWCNT and 50 wt.% GC are shown in Fig. 5i–l (uncompressed) and Fig. 5m–p (compressed). The presence of graphene in the fracture region is marked by the arrows in Fig. 5i–h. It is observed that the HBP shows uneven crack propagation relative to the BP, as seen from the fractured surface contour of the sample (Fig. 5i). Moreover, the pulled out MWCNT density and MWCNT bundles length are much less than those of BP. The graphene in the fractured regions also show folded edges, as marked by the arrow in Fig. 5k. These observations suggest that MWCNT-graphene network is loosely packed and exhibits lower van der Waals interaction and entanglement than that of MWCNT network in BP. Therefore HBP exhibits a different failure mechanism relative to BP. The compressed samples show smoother fractured surface contour (Fig. 5m), higher density of MWCNT in the pull out region (Fig. 5n), longer pulled out MWCNT (Fig. 5o), and flatten graphene sheets (Fig. 5p). Compression helps to improve the contact between MWCNT-MWCNT, MWCNT-graphene and graphene-graphene, allowing higher van der Waals interaction. It improves the mechanical performance relative to uncompressed samples (Fig. 4e,f).

**Figure 6:**
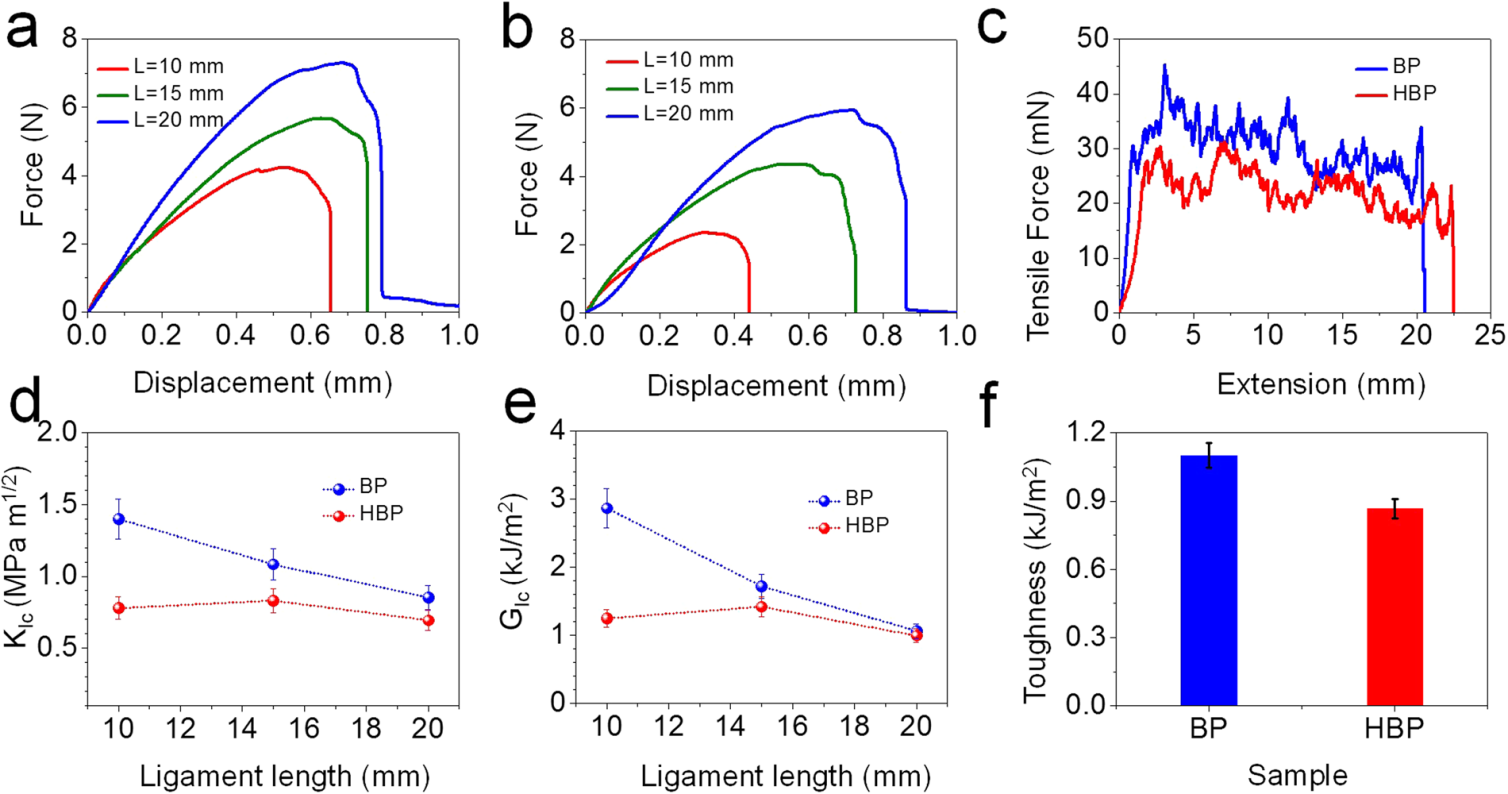
Mode I and Mode III fracture behavior: Load-displacement curves of DENT test for (**a**) BP and (**b**) HBP. (**c**) Tearing force-extension curves for BP and HBP. Comparison of (**d**) critical mode-I stress intensity factor of BP and HBP as a function of ligament length, (**e**) critical strain energy release rate (mode-I) of BP and HBP as a function of ligament length, and (**f**) tearing toughness (mode-III) of BP and HBP.

The fracture resistance of BP and HBP is characterized in Mode I using the double edge notched tension (DENT) test. The details of the tests are provided in the Methods section, and geometric parameters and dimensions of DENT specimen are provided in the Supplementary Information (Figure S8). Figure 6a,b show load-displacement curves for BP and HBP for different ligament length, $L$. It is observed that with increase in ligament length samples take more load but the strength (force/area) is insensitive to the ligament length. This insensitivity is due to stress delocalization at the ends of the ligament (Supporting Information Movies S3, S4, and Figure S9) which has been observed in BP and other fibrous network materials^62,63^. The fracture toughness, ${K}_{IC}$ and the critical strain energy release rate, ${G}_{IC}$, of BP and HBP as a function of ligament length are compared in Fig. 6d,e, respectively. The strain energy release rate is calculated using Eq. (2) in Methods section with Young’s modulus values from Table 1. BP shows an average fracture toughness of 1.11 MPa m^1/2^, whereas HBP shows an average fracture toughness of 0.76 MPa m^1/2^. The average strain energy release rate for BP and HBP is 1884 J/m^2^, and 1221 J/m^2^_,_ respectively, which are comparable to those of other reported BP and HBP^64^. The incorporation of GC into the MWCNT network lowers the overall entanglement of MWCNT. As a result, HBP shows lower fracture toughness and strain energy release rate.

The out-of-plane mode-III trouser tear tests were carried out to determine the tearing toughness of as prepared BP and HBP. The specimen geometry and photograph are provided in Supporting Information Figures S8b and S10, respectively. The effect of graphene in BP on mode-III fracture response is shown in Fig. 6c. The typical load-extension curves obtained during crack propagation showed more or less the same stick-slip fracture behavior for both BP and HBP. The stick-slip tearing behavior is typical of paper-like cellulose based materials where the crack initiates at the maximum load and arrests at lower load at regular intervals. Uddin *et al*. observed the stick-slip tearing in graphene oxide papers intercalated with CNT. The stick-slip behavior has also been observed in thermoplastic elastomers and polyvinyl alcohol gel sheets^65,66^, and in layered structures with low interfacial properties^67^. The as-prepared BP shows tearing toughness of 879 J/m^2^ whereas the as-prepared HBP shows the tearing toughness of 288 J/m^2^ (Fig. 6f). The presence of GC in HBP lowers the MWCNT network density resulting in overall lower entanglement and lower van der Waals interactions compared to BP.

## Conclusion

We successfully developed a simple, inexpensive, water-based and scalable approach to fabricate auxetic HBP of MWCNT and GC by wet-filtration-zipping. Without any chemical bonding, these self-assemblies are held together by means of entanglement of CNT and weak van der Waals force. The electrical conductivity data showed an average improvement of 35% for BP after compression. Overall, 247% improvement in the electrical conductivity is observed for HBP with 50 wt.% GC. As an application in lithium-ion batteries, HBP (50 wt.% GC and 50 wt.% MWCNT) anode shows 50% higher specific capacity and 89% lower charge transfer resistance relative to BP (100 wt.% MWCNT). In cyclic test, HBP anode shows excellent Columbic efficiency and very low cycle to cycle capacity fading. The GC content in the HBP influences the tensile performance giving a large negative Poisson’s ratio. The presence of GC flakes in the MWCNT network aids higher transverse expansion with load and results in negative Poisson’s ratio. The fracture resistance of BP and HBP is studied in two different modes, including mode I fracture using the DENT test and mode III tearing test using the trouser test. The BP shows the average fracture toughness 1.11 MPa m^1/2^ and average strain energy release rate 879 J/m^2^, whereas HBP shows the average fracture toughness 0.76 MPa m^1/2^ and the average strain energy release rate 288 J/m^2^. A green-chemistry approach, flexibility in accommodating any other 1D and 2D materials, and scalability of the method demonstrate the novelty of the work for industrial scale production.

## Methods

### Materials

Few-layer graphene flakes in a powder form (company product name ‘graphene crystals’ (GC)) were obtained from spin-off *Graphene Crystal*^*TM*^ KAUST-Saudi Arabia. The details about the GC are provided in the patent. The GC are graphene flakes with less than 10 layers containing defect-free graphene lattice (*sp*^2^ hybridized C-C bonding) over the full range of basal plane. The flake size is less than 300 µm. Multi-walled carbon nanotubes/nanostructures (MWCNT) were obtained from Applied Nanostructured Solutions, LLC. MWCNT flakes consist of bundles of aligned MWCNT. Sodium dodecyl sulfate (CH_3_(CH_2_)_11_OSO_3_Na) surfactant was purchased from Sigma Aldrich (CAS Number 151-21-3).

### Wet-filtration assembly

Schematic of wet-filtration assembly is shown in Fig. 1. In this setup, a stainless steel tank of size 19 cm × 19 cm × 10 cm (height), open on the top and bottom, is placed on the top of another metallic tank of the same size but with only the top side open. A perforated stainless steel sheet with 2 mm diameter holes and a rubber gasket are used to separate these tanks (Supporting Information Figure S7a). A filter paper purchased from Sigma Aldrich (Whatman® qualitative filter paper, grade 1 WHA1001929) is used on the top of the perforated stainless steel. This cellulose filter paper has a thickness of 180 µm and pore size of 11 µm which could easily filtrate the MWCNT and GC from the solution and hold them in the form of a thick sediment. The bottom tank is attached to the water releasing unit to discharge the filtered water so that the top tank can be filled continuously without disturbing the filtration assembly.

### Preparation of buckypaper (BP) and hybrid buckypaper (HBP)

The fabrication of BP/HBP starts with dispersing MWCNT and GC in water using the surfactant. In a typical experiment, MWCNT and GC were weighted in the range of 100 mg to 1000 mg using microbalance with the accuracy of 1 mg. The weight ratio of MWCNT and GC was varied to obtain the HBP. A BP contains only MWCNT whereas HBP contains MWCNT and GC in a different weight ratio. We also tried to obtain only GC paper but it was difficult to peel it off from the filter paper due to the weak interactions between two GC. In a glass beaker, 1 g SDS was dissolved in a 1 l water. The weighted MWCNT and GC were very easily mixed with water due to the presence of surfactant. The solution was subjected to 24 h ultra-sonication using a tip-sonicator with a 15 s ‘on’ and 10 s ‘off’ pulse of 40% amplitude and 500 W power. It was observed after 24 h that the dispersion clear black solution is obtained, moreover, the solution stays stable for the several days indicating proper dispersion of MWCNT and GC in the water (Supporting Information Figure S7b). The well-dispersed MWCNT and GC solution was poured into the number 1 tank of the wet-filtration assembly. After 12 h, the solution was filtered completely leaving behind MWCNT-GC sediment on the top of filter paper. The filtered water can be reused to prepare the next batch of samples. It saves the water without affecting the quality of BP or HBP. The sediment covered filter paper was then transferred to a heating oven on a thick, and plane aluminum sheet. The samples were heated at 90 °C for 12 h at atmospheric pressure to evaporate the water. Due to the water evaporation, the sediment zipped in a free-standing self-assembly of BP/HBP. The BP/HBP was peeled off from the filter paper. The compressed BP/HBP were obtained by compressing the BP/HBP under the optically flat stainless steel discs in a Zwick-Roell universal testing machine. The compression time and force was controlled by the sensors attached to the machine. The samples thickness was measured using Mitutoyo micrometer screw gauge with the accuracy of 1 µm.

### Sample characterization

The detailed microstructures of MWCNT, GC, and BP/HBP were characterized under the scanning electron microscope (SEM, FEI Nova Nano) and transmission electron microscope (TEM, FEI Titan G2 80-300 ST). Monochromator and image corrector were used to acquire high-resolution TEM (HR-TEM) images at 80 kV. Spectroscopic characterizations were carried out using Raman confocal spectroscopy using WITec confocal Raman spectrometer with an excitation wavelength of 532 nm (Supporting Information Figure S2a–c). Thermal behavior of the samples was characterized using thermogravimetric analysis (TGA, NETZSCH STA 449 F3 Jupiter) under inert atmosphere (N_2_ gas) (Supporting Information Figure S2d). TGA was performed from room temperature to 1000 °C at a ramp rate of 10 °C/min. The electrical resistivity of the BP/HBP with sample size of 1 cm × 1 cm was measured at room temperature using a four point configuration (Ecopia HMS 300 Hall Measurement System) following the Van der Pauw technique.

### Electrochemical characterizations

The BP/HBP were cut as 12 mm diameter disks. The mass loading (average of 3.1 mg cm^−2^) was taken using a precision balance (Mettler Toledo MS105DU Semi-Micro Analytical Balance) with a readability of 0.01 mg. Coin cells of 2032-type were assembled using a crimping machine (MSK-110 Coin Cell Crimping Machine) inside a glovebox (MBraun MB-Labstar 1450/780). The liquid electrolyte used was 1 M LiPF_6_ in EC:EMC (1:1 vol%) with 2 wt.% FEC. A half-cell configuration was used in which a lithium metal foil serves as counter and reference electrodes. Coin cells were tested using a battery tester (Maccor Battery Test System Series 4000) inside an environmental chamber (CSZ Model MC-3 Chamber) at a constant temperature of 25 °C. Battery testing was performed using constant current charge discharge (CCCD) at various rates with a potential limit between 0.01 and 3 V vs. Li/Li^+^. The BP/HBP sheets were also cut as 5 mm disk samples to be assembled in coin cell configuration for rate-capability and cyclic voltammetry tests. Cyclic voltammetry was conducted at scan rate of 0.1 mV s^−1^ from 0.01–3.0 V vs. Li/Li^+^, using a multi-channel potentiostat/galvanostat (Princeton Applied Research PMC-1000) without iR compensation. The specific capacities were calculated by considering the mass of the whole electrodes = mass of MWCNT + mass of graphene. Electrochemical impedance spectroscopy (EIS) measurements were performed obtained using an Autolab PGSTAT302N; the acquisition of the impedance spectra was done near open circuit potential (at 1.4 V) with ac frequencies between 10^6^ Hz to 2.3 × 10^−3^ Hz and ac amplitude of 10 mV.

### Tensile tests

Tensile tests of the BP/HBP with sample size of 60 × 6 × (thickness) mm^3^ were performed in Zwick-Roell Z005 device with a 20 N load cell at ambient temperature (~20°C). The tensile load was applied at a constant crosshead speed of 200 μm/min. To reduce stress localization in the grip zone, tab made from thick adhesive tape was used. DIC technique was used for full-field measurement of strain on the specimens. White speckle patterns of acrylic paint were applied on the black surface of BP/HBP using an airbrush prior to testing. Consecutive speckle images were acquired as a function of load using a monochrome 5.0 MP camera for strain evaluation. The average engineering strain in the axial and lateral directions over the gauge length zone was evaluated using Vic-2D software. These strain values were then used to construct the engineering stress‒engineering strain curve and to evaluate the Poisson’s ratio. Further details on DIC experimental setup can be found in^58^.

### Fracture toughness tests

The mode I fracture toughness of BP/HBP was evaluated using double edge notched tension (DENT) specimen configuration. The tensile load was applied at a constant crosshead speed of 200 μm/min using the Zwick-Roell machine with a 20 N load cell at ambient temperature (~20°C). The specimen with ligament length $L$, of 10, 15 and 20 mm, which corresponds to the crack length to width ratio $a/b$, of 0.67, 0.5 and 0.33, respectively were evaluated. The specimen’s configuration and geometry are shown in Supporting Information Figure S8a. The initial cracks were made using a sharp surgical blade. The critical stress intensity factor ${K}_{IC}$, was evaluated at critical stress value ${\sigma }_{c}$, for crack propagation using Eq. (1)^68^:

1 $${K}_{IC}={\sigma }_{c}\sqrt{a}[1.12\sqrt{\pi }+0.76(\frac{a}{b})-8.48{(\frac{a}{b})}^{2}+27.36{(\frac{a}{b})}^{3}]\approx 1.12{\sigma }_{c}\sqrt{\pi a}$$

The strain energy release rate ${G}_{IC}$, was obtained by:

2 $${G}_{IC}=\frac{{K}_{IC}^{2}}{E}$$

where $E$ is Young’s modulus of the material for plane-stress condition.

### Trouser Tear tests

The trouser tear tests were performed to evaluate the resistance to tear propagation of BP/HBP. The tests were conducted on the Zwick-Roel Z005 machine with 20 N load cell at cross-head speed of 2 mm/min. The critical tearing energy ${T}_{C}$, was calculated by:

3 $${T}_{C}=2F/t$$

where *F* is the mean force over the entire ligament length; and $t$ is the thickness of the specimen. Specimens were cut to the dimensions shown in Supplementary Information Figure S8b and the initial cracks were made using a sharp surgical blade. A specimen photograph during the trouser tear test is shown in Supporting Information S10.

## Electronic supplementary material

Supplementary Information
