# Supplementary material for: A wet-filtration-zipping approach for fabricating highly electroconductive and auxetic graphene/carbon nanotube hybrid buckypaper
Source: Sci Rep. 2018 Aug 15;8:12188. doi: 10.1038/s41598-018-30009-4 (PMC6093936; doi:10.1038/s41598-018-30009-4)
Supplement: Supplementary file 1 — Supplementary Information [file 41598_2018_30009_MOESM1_ESM.docx]

Supplementary Information for

**A wet-filtration-zipping approach for fabricating highly electroconductive and auxetic graphene/carbon nanotube hybrid buckypaper**

*Shashikant P. Patole^1,2^, Muhamad F. Arif^1^, Rahmat A. Susantyoko^1^, and Saif Almheiri^1^, S. Kumar^1,^^[[1]](#footnote-1)^*


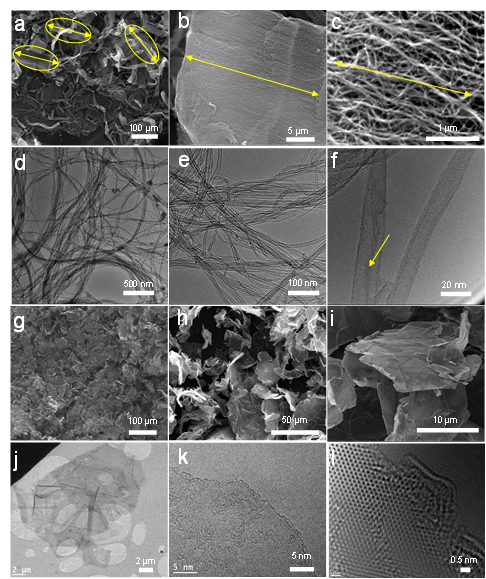


**Figure S1.** SEM images of as received multi-walled carbon nanotubes (MWCNT) powder showing a) bundled MWCNT cakes with 100-150 µm length. In the individual bundle, b) MWCNT are well aligned (the direction of alignment is marked by the arrow) and c) densely packed. TEM images of dispersed MWCNT showing d) entangled network of MWCNT, e) a 10-30 nm range diameter MWCNT, and (f) a 4, 5 and 8 walled MWCNT (arrow indicates the outer wall sharing between two CNT). SEM images of as received graphene crystal (GC) flakes powder showing g-h) crumpled GC flakes with the extension of 10-200 µm. i) The individual GC flake showing a large extension of a basal plane with wrinkled morphology. j) TEM image showing a single GC flake lying on a holey carbon grid, k) high-resolution TEM (HR-TEM) image showing the flake edge, and l) Cs aberration-corrected HR-TEM image showing honeycomb basal plane of GC flake.

Figure S1 shows scanning electron microscopy (SEM) and transmission electron microscopy (TEM) images of as-received multi-walled CNTs (MWCNT) and graphene crystal (GC) flakes. As observed in SEM images (Figure S1a-c), MWCNT are in a bundled or cake form with the individual cake ranging 110-160 µm in height and several 10-100 µm in thickness. It should be noted that the present MWCNT are synthesized by a continuous atmospheric CVD system with a glass fiber fabric substrate[^1^](#_ENREF_1). Catalyst particles were dip coated on to the glass fiber fabric prior to loading the fabric into the CVD growth chamber. After the MWCNT growth, the MWCNT cakes are separated from the glass fiber fabric by shearing. Therefore aligned MWCNT network in the growth direction can be seen in the individual cake (Figure S1b). Within the individual cake, the MWCNT are densely packed and bundled. Therefore it is important to separate the bundled MWCNT using surfactant and tip-sonication prior to the wet-filtration. The details of the MWCNT dispersion is given in the Methods section. After dispersion, MWCNT bundles are well separated allowing to form an entangled network of MWCNT (Figure S1d). The diameter of MWCNT is within 10-30 nm range (Figure S1e) with 3-10 walls (Figure S1f). It is observed that in most of the MWCNT the inner walls are intact, but the outer walls are attached to the other MWCNT making it an outstanding candidate for the buckypaper where the covalently bonded and entangled network of MWCNT is highly desirable for the electronic percolation and improved mechanical performance.

Compared to the 1D MWCNT, the 2D GC are well separated and in a crumpled form as seen in Figures S1g-i. The lateral spread of the graphene flakes in GC is in the range of 10-300 µm. Around 15 µm size graphene flake with wrinkled morphology is shown in Figure S1i. It should be noted that these GC are produced by the intercalation expansion-exfoliation process[^2^](#_ENREF_2), which gives unique wrinkled and crumpled morphology to the graphene flakes.

TEM images of a graphene flake is shown in Figure S1j. It shows an extension of 15 µm of basal plane with the wrinkled morphology lying onto the holey carbon grid. The areas near the holes are more transparent and can be used to know the exact number of layers in the graphene flakes. In such a region the high-resolution TEM (HR-TEM) shows (Figure S5k) single layer graphene. Overall the samples contain less than 10 layers graphene with less than 60% abundancy. An aberration-corrected HR-TEM is shown in Figure S1l where hexagonal honeycomb lattice with clearly distinct carbon atoms of graphene is clearly seen. It should be noted that GC are highly crystalline in nature due to its unique production procedure which does not allow the oxidation of graphitic lattice. It is also revealed in Raman and thermogravimetric analysis (TGA) (Supporting Information Figure S1). Therefore GC are also distinct from the rGO where a lot of missing carbon atoms and presence of oxygen deteriorates its crystallinity and conductivity. It is expected that the combination of 1D ultra-long MWCNT and highly-crystalline 2D GC will enhance the conductivity of combined assembly.

**Figure S2.** a) Raman spectra showing the D, G and 2D bands, b) G and D bands, and c) 2D bands of multi-walled carbon nanotubes (MWCNT), graphene crystals (GC), bucky paper (BP), compressed BP (C-BP), hybrid graphene BP (HBP) and compressed HBP (C-HBP). d) Thermogravimetric analysis (TGA) of MWCNT, GC, BP, and HBP. The heating was performed at the rate of 10 °C/min in the Nitrogen atmosphere.

Raman spectroscopy is a well-known tool to assess molecular structures of carbon materials (diamond, graphite, CNT, graphene, GO, rGO), which can be determined through their Raman spectra. The intensity ratios of the typical Raman D (vibrational mode for disordered carbon due to first-order zone boundary phonons) and G (an analogous vibration mode of *sp^2^* carbon atoms caused by the in-plane optical vibration due to degenerate zone center *E* 2g mode) peaks could be used as a metric of disorder in graphitic layers, such as edges, charged impurities, defects, and the presence of domain boundaries [^3^](#_ENREF_3). The intensity ratios of the typical Raman 2D (an analogous vibration mode of *sp^2^* carbon atoms caused by the in-plane optical vibration due to second-order zone boundary phonons) and G peaks could be used to access the number of layers in graphitic structure [^4^](#_ENREF_4). Generally, D, G and 2D peaks are found at around 1340, 1580, and 2700 cm^-1^, respectively. Figure S5a-c shows typical Raman spectra of MWCNT, GC, BP, C-BP, HBP and C-HBP. Raman shift in the range of 100 cm^-1^ to 3500 cm^-1^ is shown in Figure S5a. It is clearly seen that all the spectra contain D, G and 2D peaks confirming the graphitic carbon. Raman shift in the range of 1200 cm^-1^ to 1700 cm^-1^ is shown in Figure S5b. The as-received MWCNT show I_D_/I_G_ ratio of 0.87 confirming defective MWCNT (Supporting Information Table S1). It should be noted that unlike the conventional CNT processes that mostly focus on production of high purity CNT, the MWCNT used in this study are grown by continues CVD which allows rapid and continuous growth of CNT on a moving substrate. While the conventional CNT growth rate is typically in the order of several microns per minute, the continuous CVD can achieve a growth rate of several microns per second. As a result, the resulting MWCNT flakes are more defective than that in a conventional CNT. The MWCNT flake consists of bundles of aligned CNT (Figure S1b). Inner walls are intact, but the outer most wall that has 5 or 7 member of “C” rings which are covalently bonded with adjacent MWCNT is defective. These defective features are characterized by its highly entangled, branched, crosslinked, and wall-sharing architecture. On the contrary, GC used in this study show I_D_/I_G_ ratio of 0.06 confirming defect-free graphene. The observed D band is due to its edges structure, which is strong at the armchair edge and weak at the zigzag edge [^5^](#_ENREF_5). BP and HBP show I_D_/I_G_ ratio higher than the MWCNT. It is expected that a prolonged ultra-sonication might have caused the defects in MWCNT mainly due to shearing and breaking of MWCNT bundles. No additional defects were created due to the compression of BP and HBP. The 2D band Raman shift is shown in Figure S5c. MWCNT show symmetric broad peak centered at around 2680 cm^-1^ confirming multi-graphitic walls in CNT [^6^](#_ENREF_6). Whereas, GC show an asymmetric broad peak centered at around 2716 cm^-1^ with the intensity ratio of I_2D_/I_G_ 0.36 confirming few layers in graphene. Usually, single layer graphene is expected to have a single, sharp 2D peak, roughly two times more intense than G peak [^4^](#_ENREF_4). The shift in 2D peaks can be used to analyze mechanical strain in the graphene [^7^](#_ENREF_7). BP, C-BP, HBP, and C-HBP show shift in the 2D peaks relative to MWCNT and GC. The zipping effect during drying process might have caused the local mechanical strain on the MWCNT and GC. Apart from this, the compression doesn’t alter the 2D peak positions significantly confirming the effect of compression is limited to reduction in pore sizes and increase in density of BP and HBP.

Thermogravimetric analysis (TGA) of MWCNT, GC, BP, and HBP was carried out to know the thermal degradation behavior of BP and HBP. Figure S5d shows the TGA curves of MWCNT, GC, BP, and HBP. MWCNT show initial 5 wt.% mass loss at 110 °C due to the removal of adsorbed moisture in the samples. MWCNT start degrading above 600 °C giving 25 wt.% mass loss up to 1000 °C. The total mass remained at 1000 °C is 70 wt.% which are the unburned graphitic structure and impurities in MWCNT. Compared to MWCNT, GC show 2 wt.% initial mass loss up to 110 °C due to the removal of moisture and further 5 wt.% mass loss up to 600 °C due to the burning of amorphous carbon. The mass remained at 1000 °C is 85 wt.% which corresponds to the unburned graphitic structures. The total mass remained at 1000 °C in GC is higher than MWCNT. The higher defects in MWCNT, as revealed by the Raman spectra, act as weak oxidation centers during heating [^6^](#_ENREF_6). Compared to MWCNT, BP shows different thermal degradation behavior. Apart from initial 1.3 wt.% mass loss, BP starts degrading above 160 °C and lose 15 wt.% till 270 °C. This mass loss could be attributed to the degradation of sodium dodecyl sulfate (NaC_12_H_25_SO_3_) which is used as a surfactant for the MWCNT dispersion. Further, the impurities left behind from the SDS burned up to 600 °C contributing additional 5 wt.% mass loss. The mass left at 1000 °C is 67 wt.% which could be attributed to the unburned graphitic carbon and impurities. HBP shows a similar trend of thermal degradation up to 270 °C to that of MWCNT. In both the cases, the amount of SDS used for the dispersion was same, which gives a similar trend in the TGA curve. Afterward, up to 600 °C, the mass loss is 15 wt.%. The mass left at 1000 °C is 74 wt.% contribution from unburned graphitic carbon and impurities. The higher mass left at 1000 °C clearly demonstrates that HBP contains a higher amount of defect-free graphitic carbon from GC. It also shows that HBP is thermally more stable than BP due to the presence of defect-free graphene in it.

**Table S1:** Raman peak positions, intensity and intensity ratios of multi-walled CNT (MWCNT), graphene crystals (GC), buckypaper (BP), compressed BP (C-BP), hybrid BP (HBP) and compressed HBP (C-HBP).

| Sample | D-peak | | G-peak | | 2D-peak | | Intensity ratio | |
| --- | --- | --- | --- | --- | --- | --- | --- | --- |
|  | Position (cm^-1^) | Intensity | Position (cm^-1^) | Intensity | Position (cm^-1^) | Intensity | I_D_/I_G_ | I_2D_/I_G_ |
| MWCNTs | 1344 | 0.87 | 1574 | 1 | 2678 | 0.47 | 0.87 | 0.47 |
| GCs | 1348 | 0.06 | 1581 | 1 | 2716 | 0.36 | 0.06 | 0.36 |
| BP | 1345 | 1 | 1587 | 0.8 | 2690 | 0.4 | 1.25 | 0.50 |
| C-BP | 1346 | 1 | 1583 | 1 | 2685 | 0.44 | 1.00 | 0.44 |
| HBP | 1347 | 1 | 1581 | 0.91 | 2691 | 0.42 | 1.10 | 0.46 |
| C-HBP | 1348 | 1 | 1582 | 0.91 | 2687 | 0.38 | 1.10 | 0.42 |

**Figure S3:** Effect of MWCNT weight on the thickness of BP. It was observed that BP with thickness less than 25 µm is difficult to be separated from the cellulose filter paper.

**Figure S4:** A proposed continuous hybrid buckypaper (HBP) manufacturing route via wet-filtration-zipping method combing existing paper manufacturing technology. CNT and graphene (or other 2D materials) are dispersed in a 1) water tank using 2) tip sonication. The well-dispersed slurry is 3) spread over 4) the filter paper rolling over 9) the mesh. 7) The suction helps to drain the water and allows a uniform sediment of CNT/graphene onto a filter paper. The roller 4, 5, 6, and 8 help to keep the process running continuously. The drained sediment is fed to the 10) drying unit with 11) heater, where sediment is zipped and compressed into the HBP. The HBP and cellulose filter paper are separated by 12) a cleavage unit and collected separately on to the rolling wheel 13, and 14, respectively.

**Figure S5:** Effect of pressure on the thickness of HBP. The given pressure is applied for the 30 min and the thickness was measured after 1 hour to avoid the effect of elastic deformation.

**Figure S6:** Photographs showing buckypaper and hybrid buckypaper.

**Figure S7:** Photographs showing (a) a wet-filtration assembly; and (b) MWCNT and GC well-dispersed solution.

**Figure S8:** Geometric dimensions of (a) double edge notched tension (DENT) specimen, and (b) trouser tearing test specimen. All dimensions are in mm.

**Figure S9:** Engineering strain field of a) buckypaper (BP), and b) hybrid buckypaper (HBP) obtained from the digital image correlation (DIC) analysis in double edge notched tension (DENT) test. The strain field near the ends of ligament has the highest intensity.

**Figure S10:** A photograph showing loading of a hybrid buckypaper HBP specimen in a trouser tearing test.

**References**

1 Shah, T. K. *et al.* Carbon nanostructures and methods of making the same. WO 2014052664 A1 (2013).

2 Patole, S. P. & Costa, P. M. F. J. Expansion and exfoliation of graphite to form graphene. WO 2017125819 A1 (2017).

3 Saito, R., Hofmann, M., Dresselhaus, G., Jorio, A. & Dresselhaus, M. Raman spectroscopy of graphene and carbon nanotubes. *Advances in Physics* **60**, 413-550 (2011).

4 Ferrari, A. C. *et al.* Raman spectrum of graphene and graphene layers. *Physical review letters* **97**, 187401 (2006).

5 Ferrari, A. C. Raman spectroscopy of graphene and graphite: disorder, electron–phonon coupling, doping and nonadiabatic effects. *Solid state communications* **143**, 47-57 (2007).

6 Lehman, J. H., Terrones, M., Mansfield, E., Hurst, K. E. & Meunier, V. Evaluating the characteristics of multiwall carbon nanotubes. *Carbon* **49**, 2581-2602 (2011).

7 Ni, Z. *et al.* Raman spectroscopy of epitaxial graphene on a SiC substrate. *Physical Review B* **77**, 115416 (2008).

1. ^1^Department of Mechanical and Materials Engineering, Khalifa University of Science and Technology, Masdar Institute, Masdar City, P.O. Box 54224, Abu Dhabi, UAE. ^2^Department of Physics, Khalifa University of Science and Technology, P.O. Box 127788, Abu Dhabi, UAE.

   E-mail: s.kumar@eng.oxon.org [↑](#footnote-ref-1)
